# Patient and public involvement in the development of health services: Engagement of underserved populations in a quality improvement programme for inflammatory bowel disease using a community‐based participatory approach

**DOI:** 10.1111/hex.14004

**Published:** 2024-03-03

**Authors:** Elena Sheldon, Naseeb Ezaydi, Melanie Ditmore, Olga Fuseini, Rachel Ainley, Kerry Robinson, Daniel Hind, Alan J. Lobo

**Affiliations:** ^1^ Sheffield Centre for Health and Related Research (ScHARR) The University of Sheffield Sheffield UK; ^2^ VoiceAbility London UK; ^3^ Freelance Interpreter and Roma Consultant Sheffield UK; ^4^ Crohn's & Colitis Hatfield UK; ^5^ Sheffield Inflammatory Bowel Disease Centre, Royal Hallamshire Hospital Sheffield Teaching Hospitals NHS Foundation Trust Sheffield UK

**Keywords:** health services research, inflammatory bowel disease, patient and public involvement, quality improvement, underserved groups

## Abstract

**Introduction:**

Involving people with lived experience is fundamental to healthcare development and delivery. This is especially true for inflammatory bowel disease (IBD) services, where holistic and personalised models of care are becoming increasingly important. There is, however, a significant lack of representation of underserved and diverse groups in IBD research, and there are significant barriers to healthcare access and utilisation among minority groups in IBD. IBD centres need to be aware of these experiences to address barriers via service changes, improve interactions with local communities and promote meaningful engagement for improved health outcomes.

**Methods:**

A pragmatic community‐based approach was taken to engage with leaders and members of underserved groups across 11 workshops representing Roma, Afro‐Caribbean, people of African descent and the wider black, Asian and minority ethnic (BAME) communities, Muslim women, refugee community members, deprived areas of South Yorkshire, LGBTQ+ and deaf populations. Thematic analysis of field notes identified patterns of attention across the community groups and where improvements to services were most frequently suggested.

**Results:**

Findings demonstrated several barriers experienced to healthcare access and utilisation, including language accessibility, staff attitudes and awareness, mental health and stigma, continuity of support, and practical factors such as ease of service use and safe spaces. These barriers acted as a lever to co‐producing service changes that are responsive to the health and social care needs of these groups.

**Conclusions:**

Engaging with people from a range of communities is imperative for ensuring that service improvements in IBD are accessible and representative of individual needs and values.

**Patient or Public Contribution:**

Local community leaders and members of community groups actively participated in the co‐design and development of improvements to the IBD service for a local hospital. Their contributions further informed a pilot process for quality improvement programmes in IBD centres.

## 1 INTRODUCTION

It is widely understood that people with chronic illness should be involved in their care to support healthy living.^1^ This holds true for people with inflammatory bowel disease (IBD), lifelong conditions that include Crohn's disease (CD) and ulcerative colitis (UC). IBD symptoms are often unpredictable, debilitating and significantly impact a person's quality of life and well‐being. Patient and Public Involvement (PPI) is therefore becoming increasingly important in IBD healthcare development and delivery^2^, ^3^, ^4^, ^5^ and is an integral part of a continuously learning health system.^6^ Clinical services are moving towards holistic, more personalised models of care which consider an individual's wider context and needs.^1^

IBD services have reported examples of PPI in quality improvement initiatives where people with IBD have informed decision‐making and the co‐design of service developments.^7^ Despite these efforts, the experience of harm for people with IBD and their families has been described.^8^, ^9^ There is a significant lack of representation of underserved and diverse groups in IBD research,^10^, ^11^, ^12^ and there are substantial differences in healthcare access and utilisation among minority groups in IBD.^13^, ^14^ Socioeconomic factors such as resources and access to facilities may drive these differences,^13^ but further research is needed to confirm this. This represents an important gap in our understanding of how to deliver clinically effective and equitable IBD services.

There is also uncertainty around how PPI exercises can be undertaken in ways that constitute real and meaningful partnerships and that involve a diversity of voices^15^, ^16^, ^17^, ^18^; this limits the extent to which people with lived experience from underserved groups are able to make a tangible difference to quality improvement projects in IBD healthcare services. Community‐based participatory action research (CBPR) describes one way in which researchers may develop collaborative partnerships working with the patient partners, families and carers for whom the research will ultimately benefit.^19^ Core principles of this approach may be extended to PPI in a community setting, with a view to building relationships with patient partners and families in underserved local communities.^20^ A community‐based participatory action approach to PPI could provide an opportunity to identify health priorities for underserved groups, challenge assumptions about the patient experience and co‐create knowledge to drive forward person‐centred service developments.^21^

A CBPR approach to PPI further aligns with key aspects of policy, such as NHS England's Core20PLUS5^22^ and NHS 5 Year Forward View^23^ that advocate for a more inclusive PPI process to address health inequalities. These national health policies that underpin successful PPI have the potential to foster joint ownership in the co‐design and evaluation of health services. Our work sought to develop and pilot an inclusive, CBPR approach to PPI in the context of IBD health service development.

*A*vailable *W*ell‐defined data on *A*ctivity & *R*esults *E*mpowering people with *IBD* (AWARE‐IBD) is a quality improvement programme led by people with IBD funded by The Health Foundation (https://doi.org/10.17605/OSF.IO/H7FCP) that aims to empower people with IBD by making improvements to their care based on what matters to them. A key aim of the project was to engage people with IBD, their families and people in the local community in the co‐creation of changes to the IBD service. The PPI objectives were to understand and raise awareness of the factors underpinning the differences in healthcare access and utilisation by exploring the experiences of patient partners and families from underserved groups and what matters most to them. Learnings fed directly into the quality improvement programme to allow prioritisation of changes to the IBD service and to identify opportunities for further collaboration. Findings also fed into the co‐production of an IBD Toolkit aiming to support all people with IBD in clinical encounters by addressing the barriers identified in this research.

## 2 MATERIALS AND METHODS

### 2.1 Study design

A community‐based participatory approach to PPI was piloted as part of the AWARE‐IBD quality improvement programme. Specifically, this study applied the principles and tools of CBPR to develop person‐centred changes to the IBD service at Sheffield Teaching Hospitals NHS Foundation Trust (a single UK referral centre). Key members of the AWARE‐IBD project team (E. S., A. J. L. and K. R.) worked in partnership with a patient advocacy and involvement service, who have extensive experience working with underserved groups, to plan and deliver the PPI meetings (VoiceAbility, London, UK; https://www.voiceability.org/). A pragmatic and flexible approach was adopted, which evolved during the project based on feedback from community leaders and groups—adapting to the individual needs of diverse community groups.

The AWARE‐IBD programme used an evidence‐based clinical microsystems methodology delivered by the Sheffield Microsystems Coaching Academy^24^ to trial and implement service changes. Findings are reported in line with the GRIPP2 Short Form checklist^25^ (File S1). We described our findings in terms of the common barriers to healthcare access and utilisation amongst underserved groups and the co‐designed service changes that were trialled as part of the AWARE‐IBD programme.

### 2.2 Sampling and participants

Purposeful sampling was undertaken to identify persons from local community organisations and groups to participate in meetings as PPI contributors. Our approach was based on NHS England's (2021) Core20PLUS5, targeting the 20% most deprived people (based on the national Index of Multiple Deprivation; IMD),^26^ as well as ethnic minorities and those with other protected characteristics defined by the Equality Act 2010.

#### 2.2.1 Community leader engagement

Initial engagement was made with local community leaders who provided expertise on the logistics of working with their respective community group. These represented the Roma community, Afro‐Caribbean, people of African descent and the wider black, Asian and minority ethnic (BAME) community. Members of the AWARE‐IBD project team and VoiceAbility (E. S., N. E. and M. D.) contacted community leaders by email and arranged further engagement opportunities with members of the community group who were interested and willing to contribute.

#### 2.2.2 Community group engagement

Four community groups were available: 'Healthy Her' (a Muslim women's group); ShipShape community hub (representing BAME and refugee community members); Deep End PPI Group (representing people from deprived areas of South Yorkshire and ethnic minorities) and a local Roma community group (representing Romani people). Within those community groups, at least one contributor had a diagnosis of IBD and/or another long‐term health condition or was a family member, friend or carer of someone with IBD. Three people with IBD who identified as LGBTQIA+ and one deaf individual with IBD who were active participants in the AWARE‐IBD project volunteered for individual consultations with the team (E. S. and A. J. L.). These individuals contacted the project team by email in response to an advert placed in the AWARE‐IBD project newsletter, flyers and social media platforms.

### 2.3 Meetings

In line with the overall AWARE‐IBD programme aim, PPI meetings aimed to understand what matters most to the people both within and across community groups and learn about people's experiences of health services. This led to discussions about the barriers and facilitators to accessing healthcare for people with IBD, families and carers, including key issues related to IBD services. Community groups worked together with the AWARE‐IBD project team and VoiceAbility to prioritise these touchpoints for developing service change ideas.

PPI meetings involved individual consultations and group workshops held online and face‐to‐face in community settings across South Yorkshire, United Kingdom, based on the preferences of the community. Face‐to‐face workshops took place at local community venues that were either already used on a regular basis by the group or selected in consultation with a community group leader. The number of engagement opportunities depended on the size and scope of the community groups and their availability. We used secure videoconferencing software for online PPI meetings (Google Meet). Participants were reimbursed via bank transfer for time and travel.

Visual presentation methods and open discussion were used to facilitate discussion and key outputs. Members of the AWARE‐IBD Project team who delivered or facilitated the sessions were selected based on the nature and characteristics of the community groups. For example, female‐only professionals attended the session with the Muslim women's group.

Each workshop lasted between 1 and 2 h and took place during September 2021 and September 2023. Materials included PowerPoint slides, flip charts, sticky notes and handouts printed on A4 paper. Each session began with introductions and rapport building, followed by an explanation of IBD, common symptoms and the rationale for the AWARE‐IBD quality improvement programme. We initiated an open discussion about the barriers to healthcare access and utilisation for all community groups. The meeting purpose and activities varied however, according to the timeline of the AWARE‐IBD programme. Guidance reinforced that all voices matter and that information shared should remain confidential and not be communicated outside the group. Table 1 describes the specific purpose, format and materials used for each of the PPI meetings. Field notes were taken during the meetings to capture key themes from the discussion.

**Table 1:** Overview of the purpose, format and materials.

Number of PPI meetings: 11			
PPI contributors	Purpose and activities	Format	Materials
Deep End PPI Group	Open to discussion to (1) brainstorm patient experiences of communicating with, accessing and using healthcare services; and (2) to understand the barriers and facilitators underpinning these experiences.	One face‐to‐face group workshop, May 2022	PowerPoint presentation; post‐it notes; flip chart paper; A4 visual images
Deaf community	Discussion about patient experiences of the local IBD service from a deaf person's perspective and a professional stakeholder from the Deaf Citizens Advice team and the necessary arrangements to ensure accessibility for people with hearing disability. Verbal feedback collected on service changes trialled to date and key outputs.	Two individual consultations held online by Google Meet and email, May 2022	Handout of draft documents for review
Roma community	Open discussion about 1) common themes that have come up and 2) new issues and experiences that are unique to their community. Co‐design of key outputs.	One face‐to‐face group workshop and individual consultation, both with interpreters, September 2022	Flip chart paper; post‐it notes; handouts of draft documents
LGBTQIA+	Discussion about patient experiences of the local IBD service from an LGBTQIA+ person's perspective; verbal feedback on service changes and key outputs.	Two individual consultations held online and one held face‐to‐face, September 2022	Handout of draft documents for review
Healthy Her	Written survey feedback on patient experiences; open discussion about common issues; verbal feedback on service changes; dissemination of key outputs.	One face‐to‐face group workshop, March 2023	IBD Toolkit handouts; feedback forms
ShipShape	To raise awareness of the AWARE‐IBD project; share learnings and early findings from the project; dissemination of key outputs.	Two face‐to‐face group workshops and a local event, July 2023	Banners, flyers and copies of the IBD Toolkit

Analysis was undertaken to identify common patterns of attention that were experienced across the community groups and where improvements to the service were most frequently suggested. Analysis was undertaken by two researchers (E. S. and N. E.). Field notes and information from meeting materials (such as sticky notes and flip charts) were collated into an online document for thematic analysis.

## 3 RESULTS

An Equality, Diversity and Inclusivity Plan (File S2) was developed based on feedback collected during a consultation meeting with local community leaders. Several leaders and groups discussed the importance of having feedback on the outcomes and actions that resulted from their engagement with the programme and building a continuing relationship with clinical organisations.

We held 11 PPI meetings. Table 2 illustrates the seven key themes reported both within and across these groups. Table 3 presents the collective themes, the subsequent suggestions and priorities for service improvements and related outcomes from the AWARE‐IBD quality improvement programme. Table 4 summarises the key principles of *our* pragmatic and flexible approach to PPI with underserved groups, which developed over the course of the service improvement programme.

**Table 2:** Barriers and facilitators to health service access and utilisation within different community groups.

	Community groups							
Theme	Community leaders		Deep End PPI Group	Roma community	Deaf community	LGBTQIA+	ShipShape	Healthy Her
Language accessibility	**x**			**x**	**x**		**x**	**x**
Communicating information	**x**			**x**	**x**	**x**	**x**	**x**
Staff attitudes and awareness	**x**	**x**		**x**		**x**	**x**	
Patient advocacy	**x**			**x**		**x**		**x**
Continuity of support in treatment planning				**x**		**x**		**x**
Mental health stigma and social support		**x**				**x**		
Practical factors: Service accessibility, ease of use and safe spaces	**x**	**x**		**x**		**x**	**x**	**x**

**Table 3:** Key touchpoints for service development.

Themes	Priorities and implications for service development	AWARE‐IBD service changes (service changes and outputs)
Language accessibility	Recognise interaction of language and literacy, including in deaf patients. Improved access to translated documents; improved interpreter arrangements.	IBD Toolkit,^a^ with translations and read‐aloud functions available and 'Easy Read' formats
Communication of information	Improved communication between the hospital and service users; improved access to safe spaces	IBD Education Programme in local community venues
Patient advocacy	Knowledge and awareness of a service user's rights and the process of raising a formal complaint as ways to support advocacy	IBD Toolkit co‐designed by people living with IBD that provides the knowledge, skills and confidence to self‐advocate
Staff attitudes and awareness	Awareness of cultural considerations	IBD consultation skills workshop series with the clinical team
Practical factors: Service accessibility, ease of use and safe spaces	Improving access to the IBD service; use of patient champions or service navigators; Choice of venue for service provision	Specialist IBD consultant and IBD specialist nurse clinics to improve access for priority patients and group together hospital appointments
Continuity of support in treatment planning	Providing patient‐centred care; awareness of cultural considerations	Personalised written care plan template^b^
Mental health and stigma; access to safe spaces	Improved access to mental health support	Two education sessions were delivered by a trainee health psychologist (as part of the IBD Education Programme) focusing on Mental Health and IBD, including 'Anxiety and Worry' and 'Fatigue and Pacing'

^a^ https://www.voiceability.org/support-and-help/services-in-your-area/aware-ibd

^b^ File S3

**Table 4:** Sheffield approach to patient and public engagement with underserved groups in service improvement programmes—Key principles.

Key principle	
Take advice	Take advice from communities and their leaders about how, where and when to run engagement exercises. Use the advice to be as wide‐ranging and inclusive as possible in choosing groups with which to engage.
Respectful and aware	Respecting and having an awareness of communities' values and concerns; selection of team members to attend meetings.
Flexible	Adapt arrangements: requirements may differ between communities. Be opportunistic and contribute to existing community events.
Venue	Choose a venue based on advice and community preferences. Institutional venues (NHS, university, civic) may be intimidating.
Language	Consider the use of interpreters and translation of written materials where language support is needed.
Literacy	Literacy levels may vary alongside or independent of language barriers. Adjust material accordingly.
Feedback	Provide regular feedback to communities about the outcomes and actions resulting from consulting with them.
Evolve	Adapt approaches based on experience and feedback from previous engagement.
Build lasting relationships	Each of the principles above contributes to building a continuing relationship with underserved groups.

### 3.1 Language accessibility

Language accessibility was a common barrier cited by the majority of groups, except for two (see Table 2). Poor availability of interpreting services was reported both for people who are non‐English speaking and those requiring British Sign Language (BSL) interpreters. Some people experienced consultations where no interpreter was provided. People may rely on family or friends to translate written correspondence from primary and secondary care services; understanding of clinical letters, test results, research invitations or screening kits may therefore be limited. The Roma community group reflected on how literacy levels affect their ability to use public transport for attending hospital appointments. A professional representative from the local Deaf Citizens Advice team also reflected on the potential differences in literacy levels between those who are deaf from birth compared to those with acquired hearing loss. Groups and community leaders reinforced the interaction between language accessibility and literacy, including for deaf people where the age of hearing loss critically affects literacy. Community advice may, therefore, play an important, under‐recognised and informal role in translating and explaining clinical and clerical information from clinical departments.

### 3.2 Communication of information

In patient experience frameworks,^27^, ^28^ communication can be defined as the two‐way transfer of information between the healthcare provider and individual. However groups reflected that communication is often one‐way, with healthcare providers sending written information to individuals following a consultation, with little or no discussion about what matters to them and feeling uninformed about their health condition, the care process and treatment options available to them. Community leaders reflected that they would like to see improved two‐way communication of information in a face‐to‐face community setting, as well as opportunities for people to discuss their condition and experiences more openly.

### 3.3 Patient advocacy

Community leaders offered insights into the barriers to achieving patient advocacy where marginalised groups feel they do not have the knowledge, skills or confidence to advocate for themselves. This included a knowledge of what services were available, the skills required to access that support and the confidence to participate in care processes and decisions. People living with IBD who identified as LGBTQIA+ also described barriers to patient advocacy in terms of lacking the confidence to discuss coming out to their clinician and having open discussions around sexual activity and IBD, themes which are supported by existing research.^29^, ^30^ Community groups recommended raising awareness of a service user's rights and the process of raising a formal complaint as ways to support advocacy.

### 3.4 Staff attitudes and awareness

A common theme (across four out of the seven community groups) was the attitudes, behaviour and communication of healthcare staff, including complaints of discrimination and being dismissed. Patient partners, families and carers reflected on heightened sensitivities due to their mental health and well‐being, health‐related stress and cultural contexts. Discrimination was reported in relation to deprivation, race and sexuality across community groups. Lower satisfaction deterred further utilisation of healthcare services and created mistrust. One group identified concerns for a Roma community, which included the overriding importance of family considerations in planning treatment and prioritising the impact on the present rather than the future. The issues are also nuanced, with groups describing the effects of racism or prejudice within society, between communities and also between sections of individual communities that may be seen as a single entity. Themes are also cross‐cutting in that some issues—including sexuality, language, literacy, mental health and discrimination—are clearly intertwined with the issues of deprivation and ethnicity. In addition, approaches that are area based or community based can only make assumptions about how homogeneous a population is in terms of other characteristics.

### 3.5 Practical factors—Service accessibility, ease of use and safe spaces

Five community groups reflected on the poor availability of healthcare services to those who need the service most. Barriers included long waiting lists for appointments and procedures, a lack of flexible appointment times, and difficulties with contacting healthcare providers directly. Key practical factors that affected accessibility and ease of service use included transport to the service (based on location and affordability), disabled toilet access and navigating a large hospital building. The Roma community group felt preoccupied by the financial pressures associated with healthcare; for example, the cost of travelling to appointments at large city hospitals where their main mode of transport is a private taxi. The community group shared that they feel unable to travel by public transport due to being illiterate or having limited English language proficiency. The cost implications of travelling by taxi therefore mean that poverty and deprivation present practical barriers to accessing a healthcare service. Community leaders further discussed a lack of 'safe spaces' in care settings where environments may be perceived as unwelcoming and uncomfortable for people in their community. Feeling safe and comfortable was important for having potentially sensitive discussions about what matters to them in their health and well‐being. Recommendations for future service improvements included providing female‐only spaces, recruiting a 'patient navigator' role within the communities, undertaking clinics in the community and reducing travel needs by grouping appointments.

### 3.6 Continuity of support in treatment planning

Continuity of support was an important factor when accessing health services, in particular, the integration between healthcare professionals with regard to treatment plans and care. Two community groups reflected on the lack of coordination of care between healthcare specialists with poor awareness of a person's medical history. Groups also described a perceived lack of professionalism leading to feelings of reduced trust and confidence in both the healthcare provider and institution, which meant they were less likely to utilise the service in future.

### 3.7 Mental health, stigma and social support

Participants from two groups (Deep End PPI Group and the LGBTQIA+) reflected that they were rarely asked about their mental health during a consultation and felt there was a lack of support available from both professionals and peers. People diagnosed with IBD felt that mental health was a significant part of living with the condition, especially during a symptom flare. One participant (Deep End PPI) also described stigma associated with bowel‐type symptoms that are too embarrassing or shameful to discuss with friends and family, causing further stress and anxiety. Having a space to talk about both physical and mental health symptoms amongst peers who can relate to each other's experiences was important to those community groups.

## 4 PATIENT PARTNER PERSPECTIVE

The following perspective was given by a patient partner (O. F., Roma Community Consultant): 'I am a Czech Roma living in the UK and a passionate advocate for Roma rights. I believe we need to represent our perspectives, our culture and experiences so that more people can be aware of our shared values and needs. We are the experts in our own lives, but so often we feel that we are invisible. The AWARE‐IBD project provided a safe space for people from my community to share their experiences. It's really helpful to be part of a project that not only validates our concerns but tries to raise awareness of the barriers we face and address them with real‐life changes. It is so important for researchers, policy makers and healthcare professionals to understand the Roma population, improve their engagement skills and tackle Roma inequalities'.

## 5 DISCUSSION

The aim of our PPI process was to engage people with IBD, their families and local communities from a range of underserved groups in the co‐creation of changes to an IBD service. Our approach to PPI drew upon the principles of community‐based participatory action to better understand healthcare access and utilisation based on experiences from communities. Critical touchpoints were language accessibility, barriers to patient advocacy, communication of information between the healthcare providers and service users, practical factors such as ease of service use and safe spaces, continuity of support in treatment planning and dismissive attitudes of healthcare staff. These themes represent important challenges for overcoming cultural differences and power inequalities in the design and delivery of healthcare services for marginalised groups. Previous literature has reported how socioeconomic factors such as resources and access to facilities may drive these differences in accessing healthcare services.^13^ To our knowledge, this is the first quality improvement programme in IBD which aimed to understand and raise awareness of the barriers to healthcare access and utilisation by exploring the experiences of patient partners and families from underserved groups.

The PPI engagement supported a number of important co‐designed changes to the IBD service that aimed to address issues raised by underserved groups, which were trialled as part of the AWARE‐IBD quality improvement project.

These aimed to be applicable across the clinic population but address key aspects of the issues raised by underserved communities.

Understanding the barriers to patient advocacy was a crucial element in developing the IBD Toolkit that provides people with IBD with the knowledge, confidence and skills to self‐advocate.^31^ The toolkit was made available in multiple languages with read‐aloud functions and a simplified Easy Read version to help people with learning disabilities to access and understand information easily, using pictures to support the meaning of written text^32^ (https://www.voiceability.org/support-and-help/services-in-your-area/aware-ibd). It is also helpful to people who are not fluent in English. This included information about the local IBD service, how to access support from the telephone helpline service and how to raise a complaint.

Through the purposive event organisation, people with IBD and community groups contributed to co‐design of a personalised written care plan template to facilitate continuity of treatment planning and personalised care (File S3).

We co‐designed an IBD Education Programme to improve communication of information between the service and people with IBD, delivered in a supportive community setting across eight face‐to‐face sessions at local venues. People with IBD from underserved groups (including those living in deprived areas of Sheffield and/or BAME ethnicities) were given prioritised access to these sessions. These included two sessions dedicated to mental health and IBD, including anxiety, stress, fatigue and pacing. The sessions aimed to encourage conversation between peers and create a sense of support and connection and a safe space to discuss sensitive topics among people with lived experience, families and carers.

Staff training on cultural sensitivity and potentially sensitive topics was recommended. This fed into a workshop series with the Sheffield IBD clinical team to explore consultation skills and inform a shared approach to IBD consultations, including fostering equality and inclusivity.

These service changes will be formally evaluated as part of the AWARE‐IBD Project's embedded mixed‐methods research evaluation.

This provides a pilot process for conducting high‐quality PPI with marginalised groups in an IBD quality improvement programme. Embedding the input of underserved community groups throughout all stages of the quality improvement process ensured that the IBD service responded to identified needs and facilitated ways to maximise access and use of the service. A nonjudgemental approach that fosters a relationship of trust and confidentiality was important for achieving these outcomes. Genuine and meaningful engagement also supported sustainability in the long term for PPI engagement.

Positive feedback from the community groups provided confidence that our approach has relevance and utility for other quality improvement programmes. We recommend that underserved groups may be encouraged to participate in IBD‐related research by involving key members of their community (such as community leaders) and investing in meaningful engagement and establishing a genuine rapport. Purposive sampling methods and building long‐term relationships with community leaders contributed to our heterogeneous sample in terms of ethnicity, sexuality and socioeconomic backgrounds.

Whilst we were able to reach community groups that are typically underrepresented in IBD research and quality improvement, we were not able to represent all communities, such as those with learning disability, multiple disabilities or comorbidities. Those contacted did not want to participate or were unable to do so due to the acuity of their condition. We did not explore further how to deal with these challenges to participation and its impact on our PPI approach. As such, their voices remain unheard in the AWARE‐IBD Project. Future initiators of community‐based approaches to PPI should consider the barriers to participation for these groups and recommend solutions to facilitate involvement.

Another limitation is that in the relatively short timeframe of the AWARE‐IBD project, we were not able to address all touchpoints identified by the community groups. For example, providing female‐only safe spaces or recruiting a 'patient navigator' was not within the remit of the project in terms of timeframe and available funding. Future quality improvement programmes in IBD should, therefore, aim to implement service changes that target these particular barriers to access and utilisation. More targeted approaches in service changes, including the education programme, clinic arrangements and publicity for the toolkit supporting people in consultations, would also add to the impact of our engagement exercise. We would also encourage healthcare providers to draw upon their local community groups' experiences and insights for new service development ideas. Building meaningful and genuine relationships with community leaders further allows for making sustainable change to IBD services, as well as fostering empowerment of underserved community voices. Research teams also have an important role in involving underserved representatives across all stages of IBD research studies and ensuring meaningful input throughout.

## 6 CONCLUSION

Patient involvement is fundamental to partnership between IBD services and people with lived experience. Our findings demonstrated several barriers experienced by underserved groups in healthcare access and utilisation. Engaging with people from a range of communities and backgrounds was imperative for ensuring that service improvements were accessible, relevant and representative of patient needs and values. However, this requires an approach that is flexible and informed by the needs of local groups and their leaders. Our findings acted as a lever to co‐producing service changes that are responsive to the health and social care needs of these groups. This paper provides a pilot process for quality improvements in IBD centres that aligns with national policy and key legislation (Core20PLUS5).

## AUTHOR CONTRIBUTIONS

**Elena Sheldon**: Conceptualisation; investigation; writing—original draft; writing—review and editing; data curation; supervision; resources; project administration; formal analysis; visualisation; validation; methodology; software. **Naseeb Ezaydi**: Writing—review and editing; project administration; formal analysis; data curation; methodology; investigation; writing—original draft. **Melanie Ditmore**: Writing—review and editing; methodology; resources. **Olga Fuseini**: Writing—review and editing; methodology; resources; investigation. **Rachel Ainley**: Writing—review and editing; funding acquisition. **Kerry Robinson**: Writing—review and editing; methodology; investigation. **Daniel Hind**: Writing—review and editing; funding acquisition; conceptualisation; supervision. **Alan J. Lobo**: Writing—review and editing; funding acquisition; conceptualisation; investigation; methodology; supervision; validation.

## CONFLICT OF INTEREST STATEMENT

The authors declare no conflict of interest.

## ETHICS STATEMENT

Wales Research Ethics Committee (REC) 3 reference granted a favourable opinion (21/WA/0264) for the AWARE‐IBD Project. London—Riverside Research Ethics Committee (REC) reference granted a favourable opinion (20/PR/0974) for the AWARE‐IBD Project as a quality improvement programme, including a nested research evaluation. This work should be considered as Patient and Public Involvement, not research. It was the determination of the sponsor that whilst we can publish on a PPI exercise, the people who took part in our workshops were not research participants and worked in a service improvement (not research) paradigm. We therefore did not collect informed consent from community members. We felt that a formal research approach may alter the nature of the interaction with the professional team and the information that was shared.

## Supporting information

Supporting information.

Supporting information.

Supporting information.

## Data Availability

The data that support the findings of this study are available on request from the corresponding author. The data are not publicly available due to privacy or ethical restrictions.

## References

[1] Greenhalgh T . Patient and public involvement in chronic illness: beyond the expert patient. BMJ. 2009;338:b49. 10.1136/bmj.b49 19223339

[2] Palmer VJ , Weavell W , Callander R , et al. The participatory zeitgeist: an explanatory theoretical model of change in an era of coproduction and codesign in healthcare improvement. Med Humanit. 2019;45(3):247‐257. 10.1136/medhum-2017-011398 29954854 PMC6818522

[3] Batalden P . Getting more health from healthcare: quality improvement must acknowledge patient coproduction—an essay by Paul Batalden. BMJ (Online). 2018;362:k3617. 10.1136/bmj.k3617

[4] Batalden M , Batalden P , Margolis P , et al. Coproduction of healthcare service. BMJ Qual Saf. 2016;25(7):509‐517. 10.1136/bmjqs-2015-004315 PMC4941163 26376674

[5] Ezaydi N , Sheldon E , Kenny A , Buck ET , Weich S . Service user involvement in mental health service commissioning, development and delivery: a systematic review of service level outcomes. Health Expect. 2023;26(4):1453‐1466. 10.1111/hex.13788 37292036 PMC10349231

[6] Institute of Medicine. Patients Charting the Course: citizen engagement and the learning health system: workshop summary. National Academies Press; 2011. 10.17226/12848 22514810

[7] Fofaria RK , Barber S , Adeleke Y , et al. Stratification of inflammatory bowel disease outpatients by disease activity and risk of complications to guide out‐of‐hospital monitoring: a patient‐centred quality improvement project. BMJ Open Qual. 2019;8(3):e000546. 10.1136/bmjoq-2018-000546 PMC6683110 31428704

[8] Tsai L , Nguyen NH , Ma C , Prokop LJ , Sandborn WJ , Singh S . Systematic review and meta‐analysis: risk of hospitalization in patients with ulcerative colitis and Crohn's disease in population‐based cohort studies. Dig Dis Sci. 2022;67(6):2451‐2461. 10.1007/s10620-021-07200-1 34379220 PMC8831664

[9] McKenna NP , Bews KA , Hanson KT , Mathis KL , Lightner AL , Habermann EB . P089 racial disparities in hospital presentation and outcomes after colectomy for ulcerative colitis. Gastroenterology. 2019;156(3):S60‐S61. 10.1053/j.gastro.2019.01.150

[10] Crohn's & Colitis UK. Representation Matters [Internet]. 2023. Accessed September 21, 2023. Available from: https://crohnsandcolitis.org.uk/news-stories/news-items/representation-matters

[11] Sedano R , Hogan M , McDonald C , Aswani‐Omprakash T , Ma C , Jairath V . Underrepresentation of minorities and lack of race reporting in ulcerative colitis drug development clinical trials. Inflamm Bowel Dis. 2022;28(8):1293‐1295. 10.1093/ibd/izab362 35078220

[12] Pathiyil MM , Jena A , Venkataramana Raju AK , Omprakash TA , Sharma V , Sebastian S . Representation and reporting of diverse groups in randomised controlled trials of pharmacological agents in inflammatory bowel disease: a systematic review. Lancet Gastroenterol Hepatol. 2023;8:1143‐1151. 10.1016/S2468-1253(23)00193-0 37832569

[13] Tandon P , Chhibba T , Natt N , Singh Brar G , Malhi G , Nguyen GC . Significant racial and ethnic disparities exist in health care utilization in inflammatory bowel disease: a systematic review and meta‐analysis. Inflamm Bowel Dis. 2023;(Mar 28):izad045. 10.1093/ibd/izad045 36975373

[14] Beauchamp MLH , Amorim K , Wunderlich SN , Lai J , Scorah J , Elsabbagh M . Barriers to access and utilization of healthcare services for minority‐language speakers with neurodevelopmental disorders: A scoping review. Front Psychiatry. 2022;13:915999. 10.3389/fpsyt.2022.915999 36090362 PMC9453304

[15] Ocloo J , Garfield S , Franklin BD , Dawson S . Exploring the theory, barriers and enablers for patient and public involvement across health, social care and patient safety: a systematic review of reviews. Health Res Policy Syst. 2021;19(1):8. 10.1186/s12961-020-00644-3 33472647 PMC7816359

[16] Striving for diversity in research studies. N Engl J Med. Published online September 13 2021;385:1429‐1430. 10.1056/NEJMe2114651 34516052

[17] Martin GP , Finn R . Patients as team members: opportunities, challenges and paradoxes of including patients in multi‐professional healthcare teams. Sociol Health Illn. 2011;33(7):1050‐1065. 10.1111/j.1467-9566.2011.01356.x 21668454

[18] Rutter D , Manley C , Weaver T , Crawford MJ , Fulop N . Patients or partners? Case studies of user involvement in the planning and delivery of adult mental health services in London. Soc Sci Med. 2004;58(10):1973‐1984. 10.1016/S0277-9536(03)00401-5 15020013

[19] Jull J , Giles A , Graham ID . Community‐based participatory research and integrated knowledge translation: advancing the co‐creation of knowledge. Implement Sci. 2017;12(1):150. 10.1186/s13012-017-0696-3 29258551 PMC5735911

[20] Kwon SC , Tandon SD , Islam N , Riley L , Trinh‐Shevrin C . Applying a community‐based participatory research framework to patient and family engagement in the development of patient‐centered outcomes research and practice. Transl Behav Med. 2018;8(5):683‐691. 10.1093/tbm/ibx026 30202926 PMC6128966

[21] Greenhalgh T , Jackson C , Shaw S , Janamian T . Achieving research impact through co‐creation in community‐based health services: literature review and case study. Milbank Q. 2016;94(2):392‐429. 10.1111/1468-0009.12197 27265562 PMC4911728

[22] NHS England . Core20PLUS5 (adults)—an approach to reducing healthcare inequalities. Published 2021. Accessed September 21, 2023. https://www.england.nhs.uk/about/equality/equality-hub/national-healthcare-inequalities-improvement-programme/core20plus5/

[23] NHS England . NHS Five Year Forward View. NHS England; 2014.

[24] Nelson EC , Godfrey MM , Batalden PB , et al. Clinical microsystems, part 1. The building blocks of health systems. Jt Comm J Qual Patient Saf. 2008;34(7):367‐378. 10.1016/S1553-7250(08)34047-1 18677868

[25] Staniszewska S , Brett J , Simera I , et al. GRIPP2 reporting checklists: tools to improve reporting of patient and public involvement in research. BMJ (Online). 2017;358:j3453. 10.1136/bmj.j3453 PMC5539518 28768629

[26] Ingram E , Ledden S , Beardon S , et al. Household and area‐level social determinants of multimorbidity: a systematic review. J Epidemiol Community Health. 2021;75(3):232‐241. 10.1136/jech-2020-214691 33158940 PMC7892392

[27] Bull C . Patient satisfaction and patient experience are not interchangeable concepts. Int J Qual Health Care. 2021;33(1):mzab023. 10.1093/intqhc/mzab023 33533407

[28] Bull C , Byrnes J , Hettiarachchi R , Downes M . A systematic review of the validity and reliability of patient‐reported experience measures. Health Serv Res. 2019;54(5):1023‐1035. 10.1111/1475-6773.13187 31218671 PMC6736915

[29] Dibley L , Duffy M . Inflammatory bowel disease health care for LGTBQIA+ patients. Lancet Gastroenterol Hepatol. 2024;9(2):100‐101. 10.1016/S2468-1253(23)00352-7 37871602

[30] Fourie S , Norton C , Jackson D , Czuber‐Dochan W . ‘These discussions aren't happening’: experiences of people living with inflammatory bowel disease and talking about sexual well‐being with health care professionals. J Crohn's Colitis. 2021;15(10):1641‐1648. 10.1093/ecco-jcc/jjab043 33687428 PMC8495486

[31] VoiceAbility . Aware‐IBD self‐advocacy toolkit. 2023. Accessed September 21, 2023. https://www.voiceability.org/support-and-help/services-in-your-area/aware-ibd

[32] GOV.UK . Accessible communication formats. Accessed February 2, 2024. https://www.gov.uk/government/publications/inclusive-communication/accessible-communication-formats#easy-read-and-makaton

