# Supplementary material for: Patient and public involvement in the development of health services: Engagement of underserved populations in a quality improvement programme for inflammatory bowel disease using a community‐based participatory approach
Source: Health Expect. 2024 Mar 3;27(2):e14004. doi: 10.1111/hex.14004 (PMC10909615; doi:10.1111/hex.14004)
Supplement: Supplementary file 1 — Supporting information. [file HEX-27-e14004-s003.pdf]

Supplementary File 1. GRIPP 2 Short Form

Section and topic	Item	Reported on page No
Aim	Report the aim of PPI in the study	Page 4, lines 76-80
Methods	Provide a clear description of the methods used for PPI in the study. How we identified underserved groups.	Pages 4-8
Study results	Outcomes—Report the results of PPI in the study, including both positive and negative outcomes	Pages 8-15, see Table 3 for a summary of outcomes
Discussion and conclusions	Outcomes—Comment on the extent to which PPI influenced the study overall. Describe positive and negative effects	Pages 13 and 14
Reflections/critical perspective	Comment critically on the study, reflecting on the things that went well and those that did not, so others can learn from this experience	Pages 15-19
