# Supplementary material for: Patient and public involvement in the development of health services: Engagement of underserved populations in a quality improvement programme for inflammatory bowel disease using a community‐based participatory approach
Source: Health Expect. 2024 Mar 3;27(2):e14004. doi: 10.1111/hex.14004 (PMC10909615; doi:10.1111/hex.14004)
Supplement: Supplementary file 2 — Supporting information. [file HEX-27-e14004-s001.pdf]

AWARE-IBD Project

Putting people with inflammatory bowel disease (IBD) in control of their care.

Equality, Diversity and Inclusivity Plan

Project Partners:

The University of Sheffield (including EpiGenesys)
Crohn's & Colitis UK
Sheffield Teaching Hospitals NHS Foundation Trust
VoiceAbility

Equality, Diversity and Inclusivity (EDI) Plan

AWARE-IBD

Contents

1. The AWARE-IBD Project 1
2. Background and Purpose 1
3. Approach 2
4. Roles and Responsibilities 3
5. Identifying 'lesser heard voices' in Sheffield 5
6. VoiceAbility Community Outreach and Engagement 5
7. Partnerships to support EDI 5
8. Resources and Budget 8
9. Potential Challenges (the evidence base) 8
10. Adopting a non-stigmatising approach 9
11. Contacts and support 9
12. Useful resources 10
13. Appendix 11

**Current Version Approval** *(version 2.0)*			
**Role**	**Name**	**Signature**	**Date**
Chief Investigator	Alan Lobo		10.02.2023
Project Manager	Elena Sheldon		10.02.2023

1. The AWARE-IBD Project

AWARE-IBD is a three-year project funded under a new Health Foundation programme called Common Ambition (project number FR-000002444).

The aim of the 3-year project is to re-design IBD services, shaped by those who use them – people with Inflammatory Bowel Disease (IBD)s. Whilst the project is being delivered in Sheffield, the learning from this project will lead to better outcomes for people who live with Crohn's and Colitis across the UK. To do this, we will be working in partnership with:

- Crohn's & Colitis UK
- Sheffield Teaching Hospitals NHS Foundation Trust
- Sheffield Microsystems Coaching Academy
- The University of Sheffield
- EpiGenesys
- VoiceAbility

As part of the service improvement activities, STH will contact all IBD patients at Sheffield asking them to be involved at various levels:

Tier 1: A one-off 'what matters to you?' questionnaire
Tier 2: Being informed about service improvements and the opportunity to provide feedback
Tier 3: Convening a core patient group to select and design service changes

Research study: Collecting quarterly experience and outcome data online, by telephone or post. The University of Sheffield will use this data to understand whether service improvements are affecting patient experiences and outcomes. Researchers will also conduct in-depth interviews with a subset of these research participants and the IBD clinical team to understand what affects their views and response to the service improvements.

2. Background and Purpose of the EDI Plan

Both the service improvement and research activities need to consider Equality, Diversity and Inclusivity. This is because we want the project to impact the breadth of the Crohn's and Colitis community, and therefore we need to ensure our sample is not limited to patients who are the easiest to reach and support. The funder, the Health Foundation, is also very conscious of social inclusion[1] and reducing health inequalities in research outcomes, further strengthening the need for this plan.

This document aims to identify *who* we're aiming to engage with, *how* we will identify these patients and *what* we will do engage with them.

[1] Social inclusion: improving the terms of participation in social for groups that experience disadvantage, through enhancing opportunities, access to resources, voice and respect for rights (Mirzoev et al 2021)

3. Our Approach

The Project Leadership Group (PLG; Appendix 1) have a shared ambition to engage with a number of intersectionality groups, which are listed as protected characteristics under the Equality Act. These groups include:

- People from deprived wards in Sheffield
- People with limited English proficiency (including sign language users)
- Race and ethnic minorities (including BAME communities)
- Age (particularly those who are newly transferred from paediatric services)
- People with disabilities, e.g. a hearing disability
- People with learning disabilities
- Sexual orientation and gender reassignment (LGBTQ+)
- People who have disengaged with the IBD service (i.e. poor appointment attendance)
- Any other group that feels underserved by IBD care, where identity is a contributing factor.

Planning for equality, diversity and inclusivity will be done by completing this document.

Identifying *<u>who</u> we're aiming to engage with based on:*

- Protected characteristics under the Equality Act
- Feedback from the Health Foundation to prioritise people with IBD living in deprived areas of Sheffield
- Feedback from the Patient Oversight Committee to prioritise people with IBD who identify as LGBTQ+

Responding to EDI impacts will include:

- Keeping the EDI Plan up to date and reviewed throughout the AWARE-IBD project.
- Review of the cohort demographic data in monthly 'Super' PLG meetings.
- Publications and presentations
- Legacy documents (coordinated with CCUK)
- QI community for IBD e.g. the Health Foundation Q community
- Ongoing discussion with PPI members and other key contacts, e.g. Sheffield CCG

Our goal is…

For service improvement:

To talk to people with 'less heard voices', who are also dealing with one or more other barriers to accessing good IBD care, and understand how we can facilitate their engagement with IBD services and involvement in service improvement activities.

For research:

To improve the representativeness of the research sample by targeting recruiting towards under-represented groups.

4. Roles and Responsibilities

The Project Manager shall:

- Incorporate the resources and time required to execute the EDI Plan in the project budget and schedule.
- Develop, disseminate and implement the EDI Plan
- Coordinate with key contacts and support, such as Primary Care Sheffield and Sheffield CCG
- Update the lessons log at the end of each project phase
- Update the actions and issues log following each Super PLG meeting where the EDI Plan is discussed
- Involve the Patient Oversight Committee in discussions equality, diversity and inclusivity and keep members up to date on progress and impacts

The Clinical Trials Assistant (CTA) shall:

- Identify patients from key intersectionality groups, based on IMD Decile and BAME ethnicities
- Recruit inpatients during the IBD ward round
- Contact the identified patients about the research study, provide information sheets and (where needed) complete the consent procedure by telephone or face to face

The Project Leadership Group shall:

- Identify key intersectionality groups
- Support the Project Manager to develop and implement the EDI Plan
- Assess the impact of EDI-related actions on project success criteria
- Provide support with PPI activities, such as planning workshops and one-to-one sessions

VoiceAbility shall:

- Provide support in the planning and facilitation of community-based co-design sessions
- Provide advice as a patient advocacy service on how to approach patients in an open and honest way
- Ensure people with IBD from key intersectionality groups are involved in the co-design of the IBD Upskilling Toolkit and dissemination

Crohn's & Colitis UK shall:

- Lead the project, liaise with the Health Foundation and hold the PLG to account as the award holders

- Use their networks and partnerships, such as IBD UK, to share and embed learning from AWARE-IBD in services across the UK.
- Use their online platforms and networks to reach people with IBD from around the UK and invite them to PPI sessions

5. Identifying and engaging with 'lesser heard voices' in Sheffield

Who?	How?
People with IBD from deprived wards in Sheffield	The project Business Analyst at STH manages the IBD Database containing a list of people with an IBD access plan in Sheffield, i.e. those who are currently receiving care and open to the IBD Team. Patient postcodes have been mapped to electoral wards and Lower Super Output Areas (LSOAs) and then to a deprivation decile. Deprived wards include Burngreave, Southey, Firth Park, Manor Castle and Part and Arbourthorne. Those who did not respond to the invitation letters sent in October 2021 and May 2022 will be identified.<br><br>The CTA will contact patients identified through the IBD Database searches by telephone or email, based on the preferred contact preferences recorded. The CTA will introduce the AWARE-IBD Project, organise for an information sheet to be sent and then follow-up with telephone or online consent to the research study As of January 2023, this recruitment strategy has increased representation of IMD decile 1 (the most deprived) from 6.9% to 9.3% in the research cohort. IMD Decile 2 representation has increased from 8.8% to 11.2%.<br><br>The blue bar charts in graph below show the number of consented participants in the research cohort per IMD decile 1-10 as of January 2023. The orange line represents how this compares to the IBD cohort in Sheffield. Those in IMD decile 1 are still under-represented compared to the IBD cohort in Sheffield, however IMD decile 2 is well represented.

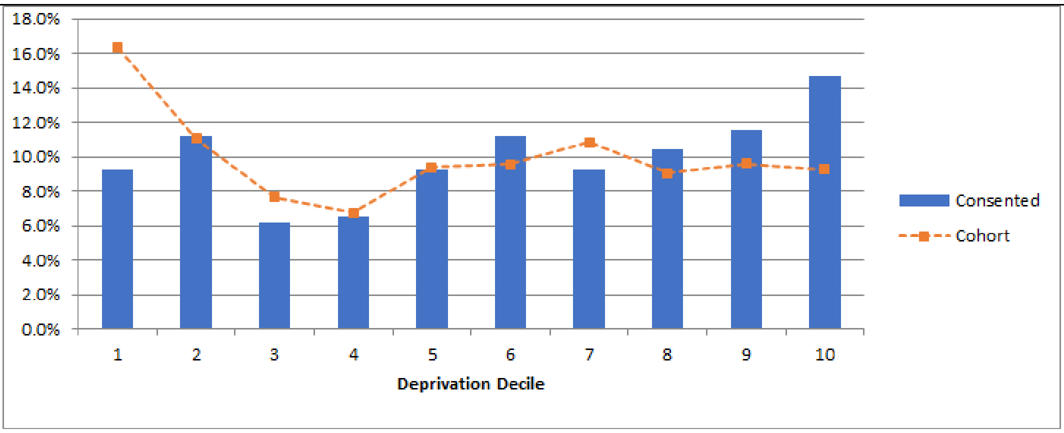

Ethnic minorities	The IBD database records patient demographic details, including ethnicity. The project Business Analyst will identify people with IBD and BAME ethnicity who did not respond to the initial invitation letters sent in October 2021 and May 2022. The CTA will contact these patients and recruit them to the AWARE-IBD Study to increase representativeness of the research sample. As of January 2023, the following ethnic categories have increased in representation:<br>• Mixed White & Black Caribbean (n=1)<br>• Mixed White & Black African (n=1)<br>• Any other mixed background (n=1)<br>• Indian (n=1)<br>• Pakistani (n=5)<br>• Any other Asian background (n=1)<br>The most significant increase in representation is for Pakistani ethnicity, increasing from 0.6 to 1.9% since the start of the study.<br>Note 6 research participants (2.3%) have a recorded ethnicity of 'No stated' meaning that the person has been given the opportunity to state their ethnic category but chose not to. . 14 research participants (5.4%) have a recorded ethnicity of 'Not known' which is where the person's ethnic category is not known.
People with hearing disability	Five patients identified at STH with hearing disability. One patient does not use BSL, so the PLG need to consider how best to communicate in this case, e.g. using the chat function or subtitles in a one-to-one session. One patient with hearing disability was contacted to provide feedback by email on the co-design of the IBD Upskilling Toolkit (led by VoiceAbility) and service improvement ideas. Changes were made to the toolkit to reflect their views and experiences, including the addition of information on how to organise a BSL interpreter for appointments. Recommendations to reintroduce the IBD Nurse email was fed back to the microsystem.

	The CI and Project Manager held a meeting with the Service Manager for Deaf Advice Team at Citizens Advice Sheffield. Feedback on how to improve access to healthcare services for people with hearing disability was collected and fed into the development of the IBD Upskilling Toolkit.
People with limited English proficiency	The project Business Analyst has conducted a database search and identified a list of IBD patients who have indicated a non-English communication preference. The top five languages are: Urdu (n=16), Polish (n=10), Arabic (n=5), Punjabi (n=4) and Somali (n=4). Note a preferred communication preference does not mean that the service user is unable to understand or speak English, but may require some support or feel more comfortable speaking in their first language.<br><br>Seven Arabic-speaking students at the University of Sheffield have volunteered to support with translation and interpretation. The PPI Flyer has been translated to Arabic. One volunteer supported the translation of the 'Journey to Diagnosis' Information Sheet and another volunteer provided interpretation support for a qualitative interview with an Arabic-speaking patient.<br><br>A panel of six interpreters at the University of Sheffield have been trained to interpret for research and translate research materials. The panel cover the following languages: Urdu/Punjabi, Pashto, Arabic, Roma/Slovak. The costs for these services are detailed in Section 8. A Punjabi/Urdu interpreter helped to contact a Punjabi-speaking patient about the Journey to Diagnosis sub-study, however they were not interested in being involved.
Learning Disability	VoiceAbility have experience of working with people with learning disability and have led the co-design of the IBD Upskilling Toolkit which includes Easy Read summaries.<br><br>A limitation of the hospital data system is that learning disability is not routinely recorded on the IBD database. This makes identifying people with IBD and learning disability difficult and will be acknowledged as a limitation of the project.
Comorbidities	The CTA will collect data from the electronic patient records on those with IBD and a diagnosed comorbidity, mental or physical. People within the same intersectionality, i.e. with the same or similar comorbidities, will be compared in the final analysis to show how this relates to the research outcomes (disease activity and patient experience).
LGBTQ+	The IBD database and patient electronic record do not routinely record LGBTQ+ information. The PLG therefore consulted with the Patient Oversight Committee to consider ways of approaching people in this group.<br><br>• Consult Stonewall and Pride in Sheffield for advice and assistance with comms as they are trusted voices and may have geographically targeted comms channels

	• Consult Lesley Dibley and the University of Greenwich who has conducted research on LBGTQ+ and IBD. This meeting took place Tuesday 29th March 2-3pm. Professor Dibley recommended the use of snowball sampling techniques<br>• Acting on this feedback, the AWARE-IBD team reached out via the project newsletter to people with IBD who are taking part in the study and/or agreed to be contacted about service improvement activities. Three individuals came forwards and 1:1 sessions with the Project Manager, IBD Nurse Specialist and patient took place. The sessions included a discussion of 'what matters to you?', accessing IBD care and feedback on the Upskilling Toolkit and service changes to ensure their views and experiences were represented.

6. VoiceAbility - Community Outreach and Engagement

VoiceAbility provide advocacy and involvement services so that people are heard in decisions about their health, care and wellbeing. As part of their partnership with the AWARE-IBD Project, VoiceAbility are reaching out to community leaders and groups in Sheffield and South Yorkshire to run a series of engagement activities. So far, the following communities have been approached:

- Roma community
- Action Collective Unity - African & Caribbean community
- SACHMA – mental health for African &Caribbean communities
- African Women's Health Group
- HealthyHer, a Muslim women's group

Workshops and 1:1 sessions with these community leaders took place in 2022 to understand their perspectives on what the main barriers are to accessing and engaging with healthcare services. In September 2022 the AWARE-IBD team led a workshop with the Roma community in Rotherham, including a 1:1 session with a non-English speaking lady with Crohn's disease and a group workshop with people diagnosed with chronic health conditions.

VoiceAbility are liaising with the community leaders to organise further engagement activities. Based on the response rate, VoiceAbility may further contact the following community organisations:

- SOAR Community Sheffield
- Darnall Wellbeing
- UK Community Foundations
- South Yorkshire's Community Foundation | Our goal is to improve the lives of local people.(sycf.org.uk)
- Health and wellbeing | NAVCA
- Community Development Team - Voluntary Action Sheffield (vas.org.uk)
- Voluntary, community and faith groups | Sheffield City Council
- Home - Traverse

As part of the engagement activities, VoiceAbility will complete an Observation Form (Appendix 2) to capture the discussion and key findings.

Engagement activities will include asking people in the community group to complete a short anonymous survey asking:

(1) What matters to you most about your healthcare?
(2) What are the main barriers you face to accessing healthcare?
(3) What support would you need to overcome these barriers to accessing healthcare?
(4) How can we improve your patient experience?
(5) Do you have a diagnosis of inflammatory bowel disease? (Crohn's disease, ulcerative colitis)

Those who complete the survey will be entered into a raffle prize drawer to win Love2Shop vouchers.

7. Partnerships to support EDI

Primary Care Sheffield

The AWARE-IBD Project links well with the Sheffield's NIHR BRC renewal which uses primary care as a mechanisms of linking researchers with patients, via primary care sites, and in turning using this mechanisms to build-in EDI in all research in Sheffield. Primary Care Sheffield (PCS) is a federation of 75 GP practices in Sheffield covering around 600,000 patients (Appendix 3). The purpose of PCS is to improve the quality of care in Sheffield, make healthcare more accessible and reduce health inequalities. The University of Sheffield will set up six Clover Group practices as Participant Identification Centres (PIC) sites to help identify 'hard to reach' patient groups. Practices will send a text message to eligible patients registered at the practice encouraging them to get in touch with the AWARE-IBD team.

Voluntary Sector Organisations

The PLG have consulted Professor Mark Gamsu, Helen Mulholland and Professor Marion Sloan who are members of the Sheffield CCG to identify and approach relevant voluntary sector organisations.

Deep End PPI Group

The Deep End PPI group are a patient participation panel who input into research studies in order to make research more accessible and inclusive. The panel were established specifically for patients living in the most deprived areas of Sheffield to allow researchers to access discussions with people from this demographic. The PPI group therefore supports local academics and researchers in Sheffield to develop research priorities that reflect the needs of the population.

The Project Manager and Chief Investigator facilitated a face-to-face workshop with the Deep End PPI Group on 23rd May 2022 to discuss barriers and enablers to participation in service improvement activities and research, with a view to understanding how barriers may be overcome. A poster was designed to summarise the findings to be presented at a subsequent celebration event (see Appendix 4).

Other recruitment methods:

- Crohn's & Colitis UK social media platforms, website and database (i.e. where people contact Crohn's & Colitis UK to share their story)
- Connect magazine
- PPI Flyer– also translated in Arabic (Appendix 5a and 5b) – placed in:
    - BAME churches and church leaders
    - Mosques and Imams
    - Food banks
    - Community groups/hubs/centres in Sheffield
    - Relevant peer led support groups
    - Advertising in public spaces like cafes, hair dressers, and community centres
    - Statutory services like GPs, social prescribers, etc. including Darnall Well Being
    - IBDesis (IBD South Asians)
    - Social media platforms, including the AWARE-IBD Twitter account, Crohn's & Colitis UK Health Services Twitter account, Crohn's & Colitis UK South Yorkshire Network Facebook account, STH's and The University of Sheffield's Facebook and Twitter accounts

8. Resources and Budget

The budget for this project does not include items related equality, diversity and inclusivity and does not include translation/interpretation services.

Funding is allocated to:

- Community meetings £3.3k per year
    - This will be used to reimburse PPI representatives and community leaders, travel costs, refreshments and room hire for community-based meetings
- VoiceAbility £2.4k total to run 'Community based co-design sessions'
- QI meetings, inconvenience fees and catering total £4k across the 3-year project
    - This will be used to reimburse patient representatives and provide refreshments for those who attend quality improvement microsystem meetings
- A funded Clinical Trials Assistant who will oversee inpatient recruitment and the targeted recruitment of under-represented patients to improve the representativeness of the research sample

Trained interpreters provided by the University of Sheffield typically charge:

- £20 per hour for interpretation, including travel and debriefing time

- £45-85 for translation of recruitment materials per document, depending on the document length

Arabic-speaking student volunteers were offered non-financial incentives including authorship on relevant publications.

9. Potential Challenges (the evidence base)

A Toolkit[2], endorsed by the NIHR, published the following guidance for increasing participation of Black Asian and Minority Ethnic (BAME) Groups in health and social care research:

Language barriers to consider

1) Questionnaire data

- Self-completion questionnaires sent to certain ethnic groups (e.g. postal surveys) often have poor response rates. Even with tailored covering letters response rates are often low because of misunderstanding/suspicion of the purpose of the research and language/ literacy issues.
- If the postal method is used, a translated sheet should be enclosed that allows respondents to request information in another language.
- Respondents who have language or literacy barriers sometimes ask family members and friends to help them to complete questionnaires. Responses may therefore not reflect the views of the target groups.
- Translated questionnaires are often not cost-effective. Translations must be checked properly (and piloted if possible) to make sure they are accurate, make sense, and that the language is not too formal or traditional.
- It should also be noted that second and third generation respondents may speak the language fluently but not read it.

2) Interviews

- 'Real time' translation in a face-to-face interview produces better response rates
- Use interviewers who are able to speak the same language as the respondents.
- Translators accompanying interviewers are possible alternatives but will require a fieldwork phase that is much longer, expensive, and less satisfactory.
- Same sex interviewing may be necessary for some groups
- Allow a minimum of 20% more time for undertaking translated interviews

[2] https://arc-em.nihr.ac.uk/clahrcs-store/increasing-participation-black-asian-and-minority-ethnic-bame-groups-health-and-social

- Interviewers must have a strong ability in both languages and be well briefed, rehearsed, supported, and quality controlled

10. Adopting a non-stigmatising approach

Trust and confidentiality are very important; people will come forward if they trust you, and you make them feel at ease. A genuine non-judgemental approach and communication with people is most important.

Representatives from the PLG will explain why this project is important, how it will benefit the individual and the community, and the potential difference it could make in the local and wider context. Emphasis will also be placed on the value that patients bring to the project as people with lived experience of IBD. This value is imperative for AWARE-IBD to be successful, ensuring that service improvement is accessible, relevant and beneficial to all patient groups and that the service changes are widely representative of patient needs and values. We will explain how bringing people from the community will add value to the project and research. Members of the PLG are conscious of potential cultural bias, stereotypes and prejudices. We will consult VoiceAbility and National Voices for advice on this.

See Useful Resources (Section 12) for VoiceAbility SOC How to run an accessible workshop

11. Contacts and Support

- Mr Jon Dixon, Sheffield GP and Primary Care Sheffield Lead
- Miss Katie Biggs, University of Sheffield - NIHR Pre-Doctoral Fellowship on inclusivity
- Dr Lucy Wasinski, STH CRIO Research Coordinator - PPI, communications and events
- Dr Liz Croot, University of Sheffield – Learning disability research
- Dr Kate Fryer – Research Associate, Management School, University of Sheffield – specialist interest in equality, diversity and inclusivity in research
- Prof Mark Gamsu – Sheffield CCG, chair of the Patient Experience, Equalities and Engagement Committee and vice chair of the Primary Care Commissioning Committee
- Prof Marion Sloan – GP and member of Sheffield CCG

12. Useful resources

- Toolkit for increasing participation of Black Asian and Minority Ethnic (BAME) Groups in health and social care: link here
- How to generate inclusive and diverse mental health patient and public involvement and engagement: link here
- VoiceAbility SOC How to run an accessible meeting
- VoiceAbility Speak Out Council Meeting Guide FINAL (for LD)
- FINAL Speak Out Council Communication Report v1
- Barriers to inclusion blog: https://shapingourlives.org.uk/2022/04/13/overcoming-the-barriers/
- Talking Mats – as a communication tool: Talking Mats | Improving communication, improving lives

Version Control

Document Version Control			
**Version**	**Date Approved**	**Approved by**	**Summary of Changes**
2.0		Elena Sheldon (Project Manager), On behalf of the PLG	• Inpatient ward recruitment (as per Non Substantial Amendment 3)<br>• VoiceAbility's community based co-design sessions<br>• PPI activities with the Deep End PPI Group and other partners supporting EDI engagement<br>• Targeted recruitment findings for people with BAME ethnicity and/or in IMD Deciles 1 and 2<br>• LGBTQ+ engagement<br>• Staff changes

Appendix 1 - The AWARE-IBD Project Leadership Group

**Name**	**Role**	**Institution**
Alan Lobo	Chief Investigator (Chair)	Sheffield Teaching Hospitals NHS Foundation Trust
Rachel Ainley	Head of Research & Evidence	Crohn's & Colitis UK
Ruth Wakeman	Director of Services, Advocacy & Evidence	Crohn's & Colitis UK
Daniel Hind	Professor of Evaluation, CTRU Lead	School of Health and Related Research, The University of Sheffield

Elena Sheldon	Study Manager	School of Health and Related Research, The University of Sheffield
Sienna A Hamer-Kiwacz	Research Assistant	School of Health and Related Research, The University of Sheffield
Agnieszka Ossowska	Trial Support Officer	School of Health and Related Research, The University of Sheffield
Samantha McCormick	Patient Microsystems Coach	Sheffield Teaching Hospitals NHS Foundation Trust
Kevin Randall	Business Analyst	Sheffield Teaching Hospitals NHS Foundation Trust
Chris Murray	Managing Director	epiGenesys
Stefan Roman	Clinical Trials Assistant	Sheffield Teaching Hospitals NHS Foundation Trust

Appendix 2 - Primary Care Sheffield

Location of GP sites in Sheffield (within Primary Care Network boundaries)

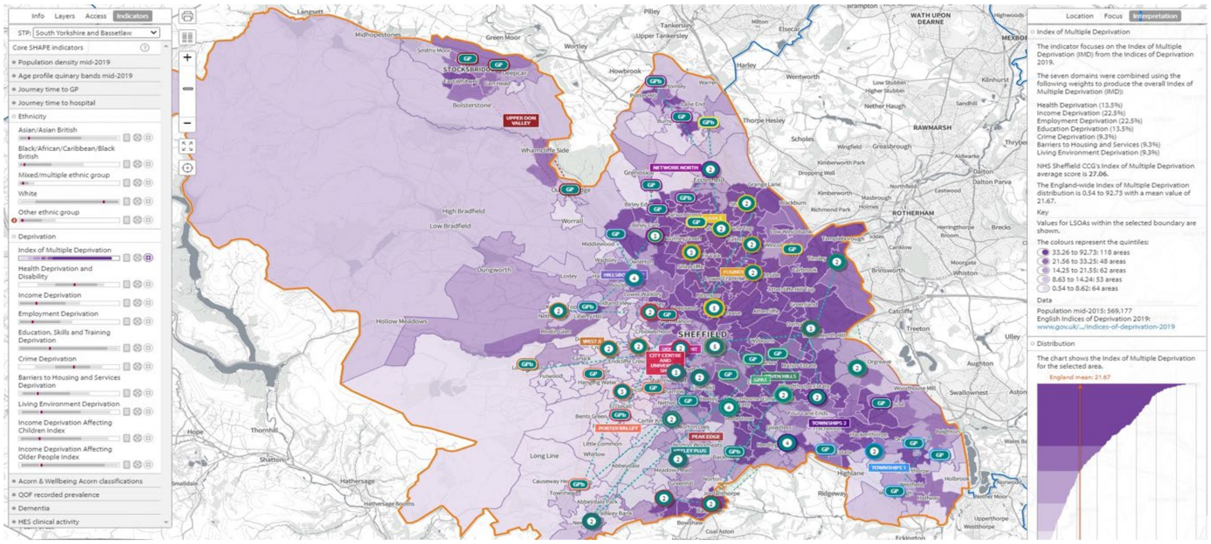

Info
Layers
Access
Indicators
STP: South Yorkshire and Bassetlaw
Core SHAPE indicators
Population density mid-2019
Age profile quinary bands mid-2019
Journey time to GP
Journey time to hospital
Ethnicity
Asian/Asian British
Black/African/Caribbean/Black British
Mixed/multiple ethnic group
White
Other ethnic group
Deprivation
Index of Multiple Deprivation
Health Deprivation and Disability
Income Deprivation
Employment Deprivation
Education, Skills and Training Deprivation
Crime Deprivation
Barriers to Housing and Services Deprivation
Living Environment Deprivation
Income Deprivation Affecting Children Index
Income Deprivation Affecting Older People Index
Acorn & Wellbeing Acorn classifications
QOF recorded prevalence
Dementia
HES clinical activity
SHEFFIELD
Location
Focus
Interpretation
Index of Multiple Deprivation
The indicator focuses on the Index of Multiple Deprivation (IMD) from the Indices of Deprivation 2019.
The seven domains were combined using the following weights to produce the overall Index of Multiple Deprivation (IMD):
Health Deprivation (13.5%)
Income Deprivation (22.5%)
Employment Deprivation (22.5%)
Education Deprivation (13.5%)
Crime Deprivation (9.3%)
Barriers to Housing and Services (9.3%)
Living Environment Deprivation (9.3%)
NHS Sheffield CCG's Index of Multiple Deprivation average score is 27.06.
The England-wide Index of Multiple Deprivation distribution is 0.54 to 92.73 with a mean value of 21.67.
Key
Values for LSOAs within the selected boundary are shown.
The colours represent the quintiles:
33.26 to 92.73: 118 areas
21.56 to 33.25: 48 areas
14.25 to 21.55: 62 areas
8.63 to 14.24: 53 areas
0.54 to 8.62: 64 areas
Data
Population mid-2015: 569,177
English Indices of Deprivation 2019:
www.gov.uk/.../indices-of-deprivation-2019
Distribution
The chart shows the Index of Multiple Deprivation for the selected area.
England mean: 21.67

Appendix 3 – VoiceAbility Community Engagement Observation Form

VoiceAbility

Community Engagement

**Community Organisation**	**Community Leader(s)**	**Date of engagement**
**Session Delivered by**	**Supported by**	**Time of engagement**

Who was the session aimed at:
Where was the session held?
Summary of the structure of the session/discussion:
Community feedback (individual/group) :
What follow up is requested/appropriate?
Possible next steps:
Were individuals receptive to completing the feedback form?

Appendix 4 – Deep End PPI Group Celebration Poster

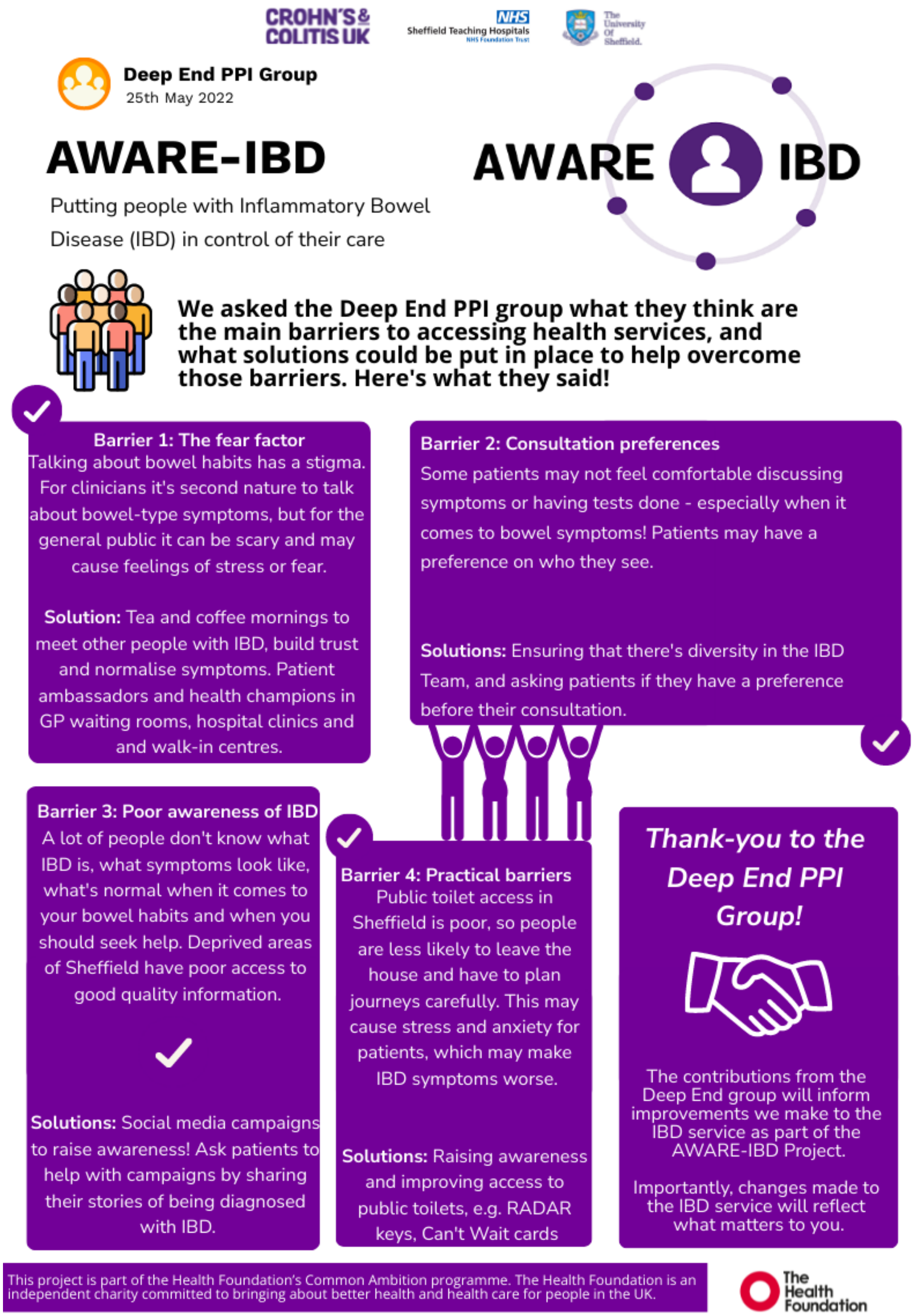

Appendix 5 PPI recruitment flyer

5a -English version

The University Of Sheffield. CROHN'S & COLITIS UK NHS Sheffield Teaching Hospitals NHS Foundation Trust

Are you living with Crohn's Disease or Ulcerative Colitis?

Please help improve services!

Crohn's & Colitis UK want to know about your experience of getting a diagnosis.

Please join other people with Inflammatory Bowel Disease (IBD) at a series of workshops.

For more information, please contact:

aware-ibd@sheffield.ac.uk

or

call 0114 222 4307

AWARE IBD

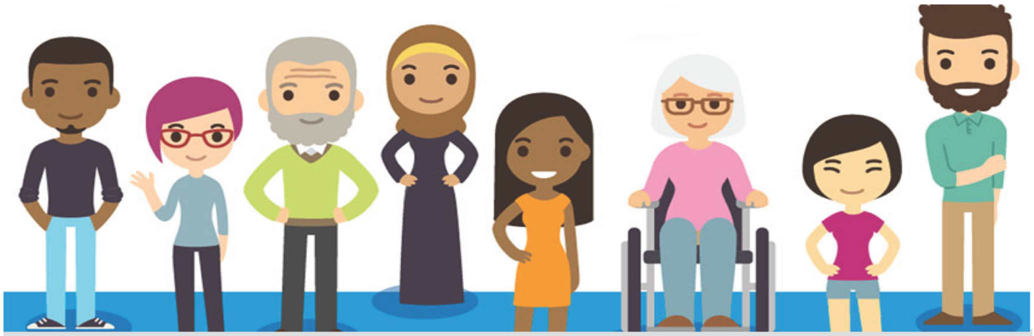

@AwareIbd

AWARE-IBD PPI Recruitment Flyer version 1, 17.01.2022 [IRAS 298930]

5b -Arabic version

The University Of Sheffield.

CROHN'S & COLITIS UK

NHS Sheffield Teaching Hospitals NHS Foundation Trust

هل لديك مرض كرون أو التهاب القولون التقرحي ؟

نرجو مساعدتنا في تحسين الخدمة

كرون و التهاب القولون التقرحي في بريطانيا ترغب بالتعرف على تجربتك مع التشخيص بهذا المرض.

الرجاء الأنضمام مع أخرين مصابين بمرض إلتهاب الأمعاء إلى مجموعه من ورش العمل.

لمعلومات اكثر،
الرجاء التواصل معنا على:
aware-ibd@sheffield.ac.uk

AWARE IBD

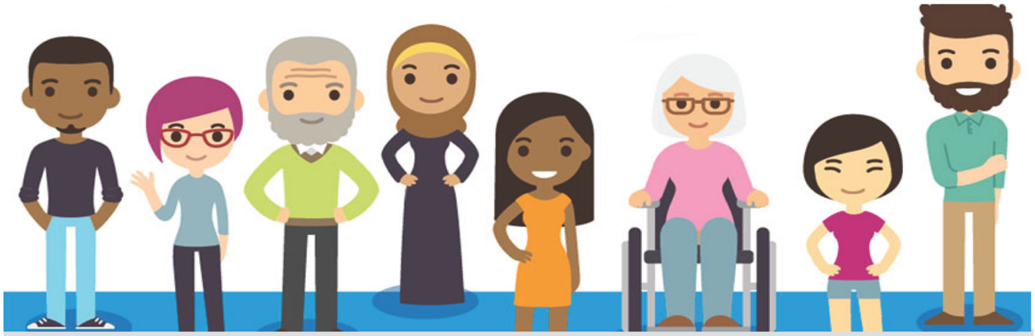

@AwareIbd

AWARE-IBD PPI Recruitment Flyer version 1, 17.01.2022
[IRAS 298930]
