# Supplementary material for: Patient and public involvement in the development of health services: Engagement of underserved populations in a quality improvement programme for inflammatory bowel disease using a community‐based participatory approach
Source: Health Expect. 2024 Mar 3;27(2):e14004. doi: 10.1111/hex.14004 (PMC10909615; doi:10.1111/hex.14004)
Supplement: Supplementary file 3 — Supporting information. [file HEX-27-e14004-s002.pdf]

Sheffield Inflammatory Bowel Disease Centre
Personalised Care Plan

NHS
Sheffield Teaching Hospitals
NHS Foundation Trust

We will use this to talk about how you are feeling and what matters to you. Knowing what matters most to you will help us to work together to live your best life. Please note that the personalised care plan is **optional.** Please let your clinician know if you do not want a personalised care plan.

Your name: ______________________ **Hospital number:** ________________ (optional) **Date:** _ _ / _ _ / _ _ _ _

My Preferred Pronouns are: _______________

Diagnosis

My diagnosis is:

Year I was diagnosed:

What is affected? Use this space to draw a diagram

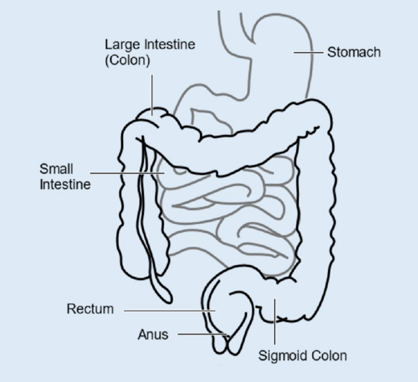

Background and Support

My IBD History

What treatment or operations I have had in the past, results from procedures, allergies or bad reactions:

What should I do if I have a flare up?

What should I do out of hours?

Contact

Who's on my IBD Care Team and how do I contact them?

My Consultant is: ______________________

My consultant's secretary is: [Name and contact details]

IBD Nurse Helpline (0114) 271 2209

Monday to Friday 9am–11am; messages picked up daily

Other useful contacts:

Infusion Appointments: (0114) 2712133
Outpatient Appointments: (0114) 2268680
Endoscopy Appointments (0114) 2713173
Capsule Endoscopy Service: (0114) 2713743
Investigations Unit: (0114) 2713156

Sources of IBD Information and Support:

Sheffield IBD Centre: https://sheffield-ibd.sth.nhs.uk/
Crohn's and Colitis UK: https://crohnsandcolitis.org.uk/
On the Crohn's and Colitis UK website you can find information on symptoms, diet, sex and relationships, drug treatments, surgery, employment and education, benefits and finances.
Sheffield IAPT (Improving Access to Psychological Therapies): https://iaptsheffield.nhs.uk/

Sheffield Inflammatory Bowel Disease Centre
Personalised Care Plan

Sheffield Teaching Hospitals
NHS Foundation Trust

How have things been going for me? Please note any issues or concerns you have about your day-to-day life and managing your IBD overall (work, home, feelings, psychological difficulties, symptoms). Please feel free to include issues that you think you might find difficult to raise otherwise.

What Matters To Me? Please tell us what is important to you. This may include, but is not limited to, your values and needs, your sexuality, gender or identity, goals for your IBD, your hopes and aspirations, future plans and support

Managing my diet, lifestyle and other health-related conditions Please use this space to record any diet, lifestyle or other health related conditions considerations that are important to you. What would you like to discuss or receive more information on?

Current treatment, next steps and review date Please note any current treatment, follow up appointments, referrals, planned investigations or surgeries. This may also include future options or alternatives to your current treatment.
